# Silver Nanoparticles Stimulates Spermatogenesis Impairments and Hematological Alterations in Testis and Epididymis of Male Rats

**DOI:** 10.3390/molecules25051063

**Published:** 2020-02-27

**Authors:** Janet Olayemi Olugbodi, Oladipupo David, Ene Naomi Oketa, Bashir Lawal, Bamidele Joseph Okoli, Fanyana Mtunzi

**Affiliations:** 1 Department of Biochemistry, Bingham University, Abuja-Keffi expressway Road, P.M.B 005 Karu, Nigeria; oketanaomiene87@gmail.com; 2 Department of Medical Bioscience, University of the Western Cape, Bellville, Cape Town 7530, South Africa; 3681075@myuwc.ac.za; 3 PhD Program for Cancer Molecular Biology and Drug Discovery, College of Medical Science and Technology, Taipei Medical University and Academia Sinica, Taipei 111, Taiwan; bashirlawal12@gmail.com; 4 Institute of Chemical and Biotechnology, Vaal University of Technology, Science Park, Private Bag x021, South Africa; okolibj@binghamuni.edu.ng; 5 Department of Chemical Sciences, Bingham University, Abuja-Keffi Expressway Road, P.M.B 005 Karu, Nigeria; 6 DIHLARE Remedy, Faculty of Applied and Computer Sciences, Vaal University of Technology, Science Park, Private Bag x021, South Africa; fanyana@vut.ac.za

**Keywords:** antioxidants, epididymis, silver nanoparticles, sperm parameters, hormones, toxicity, testis

## Abstract

The potential pharmaceutical application of nanoparticles has led to the toxicity within the male reproductive system. In the present study, the effects of silver nanoparticles (Ag-NPs) on hematological parameters, free radical generation, antioxidant system, sperm parameters, and organ histo-morphometry in male rats were investigated. Ag-NPs were produced by the reduction of silver ions, while the formation of which was monitored by UV–visible spectrophotometry. Zeta potential, transmission, and scanning electron microscopies were applied for the characterization of AgNPs. A total of 30 rats were divided into 6 groups and were sub-dermally exposed to Ag-NPs at the dosage of 0 (control), 10, and 50 mg/kg bodyweight (bw) doses for either 7 or 28 days. Ag-NP administration altered hematological indices and caused dose-dependent decreases in sperm motility, velocity, kinematic parameters, concentrations of luteinizing hormone, follicle-stimulating hormone, and testosterone. In the epididymis and testis, the concentrations of malondialdehyde and peroxide increases while superoxide dismutase, catalase, reduced glutathione, and total thiol group decreases. These findings suggest that Ag-NP triggered hormonal imbalance and induce oxidative stress in testis and epididymis; which negatively affect sperm parameters of male rats.

## 1. Introduction

Nanoparticles (NPs) are particles that are designed and produced with a dimension or size that is ≤ 100 nanometers [1]. Due to their unique physical and chemical properties such as thermal, optical and electrical, high electrical conductivity, and biological properties [2,3], NPs have found application in various fields, including biomedicine, diseases diagnosis, gene, drug delivery, fuel additives, catalyst, cosmetics, agriculture pharmaceuticals, the food industry, orthopedics, and antimicrobial therapy, etc. [2,3,4,5,6]. Despite the benefits of NPs, several shortfalls have led to potential toxicities in both humans and animals [7]. NPs have been implicated in different ailments, including pulmonary injury, hepatic and renal damage, immuno-nanotoxicity, neurotoxicity, and irreversible testis impairments [8,9].

Reproductive toxicity is increasingly becoming recognized as an important part of overall toxicology [10]. Fertility, reproduction, and fetal development are essential to the sustenance of a species, highlighting the importance of the growing public awareness of the toxicity of NPs on the reproductive system. Recent studies have indicated an increased incidence of male reproductive defects, including low sperm production in adulthood, hypospadias, cryptorchidism, and testicular cancer [11]. This increased incidence of male reproductive defects may be partly attributable to environmental contaminant exposure [10]. Thus, the wide use and environmental persistence of NPs have raised concerns about the negative impact on human health, especially on the reproductive systems and fetal health [12].

Nanoparticles have different effects on sperm cell functions either upon direct exposure under in vitro conditions or if administered in vivo [3]. The ability of NP to cross the hemato-testicular barrier has been demonstrated raising concerns about their distribution and biocompatibility at the systemic level [3].

Several studies have reported the effects of Ag-NPs on epididymis, testis, and sperm function parameters in different animal species [11,12,13]. However, the effects of NPs on oxidative stress and spermatogenesis need to be investigated on a case-by-case basis due to the administered concentration, and duration of treatment [14,15]. Therefore, the present study evaluated the effect of different doses of Ag-NP on the free radical generations, antioxidant enzymes, sperm parameters, hematological parameters, and testicular histo-morphometry in rats.

## 2. Results

### 2.1. Characterization of the Ag-NP

**Figure 1:**
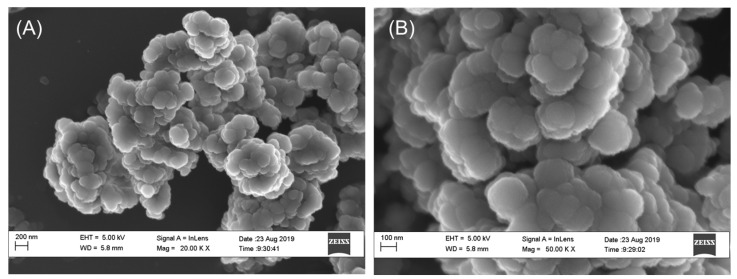
SEM micrographs of dry Ag-NPs at (**A**) 20,000×, and (**B**) 50,000×. The synthesized Ag-NPs were agglomerated and approximately spherical in shape.

**Figure 2:**
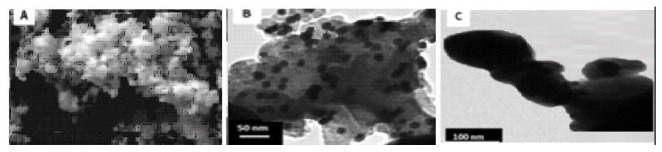
TEM micrograph of AgNPs in suspension at a working distance of (**a**) **10 nm** (**b**) **50 nm** (**c**) 100 nm.

**Figure 3:**
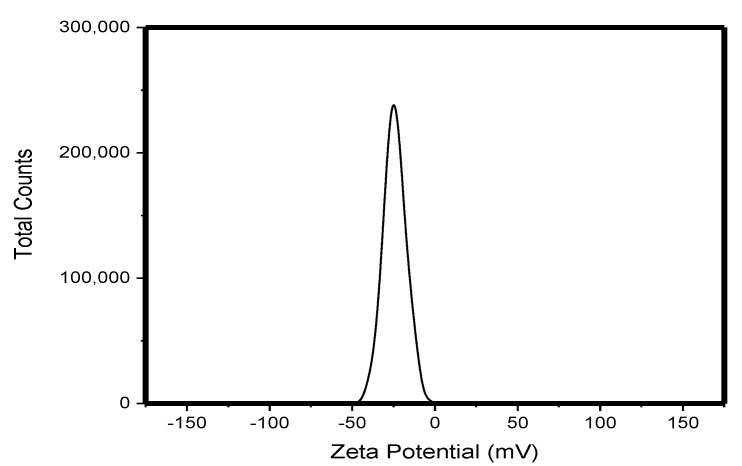
Zeta potential distribution of AgNPs.

The synthesized Ag-NPs were approximately spherical with an average particle size of 113.389 ± 22.964 nm (Figure 1) and a surface area of 7.5329 m^2^/g. The TEM micrograph of the AgNPs in suspension showed the formation of large aggregates. The suspended sizes of the Ag-NP ranged between 20–1000 nm (Figure 2a–c)). The particles exhibited distinct surface charges with a zeta potential (Figure 3) and derivative values of −18.9 mV and 7.35 mV, respectively whereas the electrical conductivity value of 0.00989 mS/cm (Figure 3). 

### 2.2. Body Weight

There were significant reductions (*p* < 0.05) in the mean body weight of rats treated for 7 and 28 days with Ag-NP (10 and 50 mg/kg bw) when compared with the control group. There were no dose-related significant differences (*p* > 0.05) in mean body weight loss of rats treated for 7 days. However, after the 28 days of treatment; 50 mg/kg bw Ag-NP caused a significantly higher weight loss than 10 mg/kg bw (Table 1).

### 2.3. Relative Organ Weight

The relative weight of epididymis and testes in rats dosed Ag-NP for 7 days were significantly (*p* < 0.05) lower than their respective control groups. The 28 days of treatment; however, caused no significant differences (*p* > 0.05) in the relative weight of the epididymis and testes between the treated groups (10 and 50 mg/kg bw) and the control groups (Table 2).

### 2.4. Hematological Parameters

In comparison with their respective normal controls, mean platelet volume (MPV), mean corpuscular hemoglobin (MCH), and mean corpuscular volume (MCV) were significantly (*p* < 0.05) lower in rats dosed 50 mg/kg bw Ag-NP for 7 days, but higher in rats dosed 10 mg/kg bw for 28 days, red blood cell (RBC), and hemoglobin (Hb) count significantly (*p* < 0.05) increase in rats dosed 10 and 50 mg/kg bw Ag-NP for 28 days while platelet (PLT) count was significantly (*p* < 0.05) higher in rats dosed 10 mg/kg bw for 7 and 28 days. There were dose-dependent decreases (*p* < 0.05) in WBC, MID%, Lym counts in rats dosed Ag-NP for 7 and 28 days. However, there were no significant differences (*p* > 0.05) in mean red blood cell distribution width (RDW-CV) and hematocrit (HCT) counts between the treated groups and the control group (Table 3).

### 2.5. Hormonal Concentrations

There were significant (*p* < 0.05) dosed dependent decreases in the hormonal concentrations of luteinizing hormone (LH), follicle-stimulating hormone (FSH), and testosterone in rats; dosed Ag-NP for 7 and 28 days (Table 4).

### 2.6. Effect of the Ag-NP on Sperm Parameters

There were significant (*p* < 0.05) dose-dependent decreases in all sperm velocity parameters (ALH, beat cross frequency, linearity, and straightness) investigated in male rats treated with 10 and 50 mg/kg bw for 7 days and those treated for 28 days when compared with the normal control rats. In respect to sperm motility, there were significant (*p* < 0.05) dose-dependent decreases in sperm total motility and progressive motility, while the sperm non-progressive motility and immobility increase significantly in a dose-dependent manner when compared with the normal control. Sperm kinematics including straight-line velocity, curvilinear velocity, and average path velocity also decreases significantly (*p* < 0.05) in a dose-dependent manner when compared with the control (Table 5, Table 6 and Table 7).

### 2.7. Testes and Epididymis Biochemical Parameters

**Figure 4:**
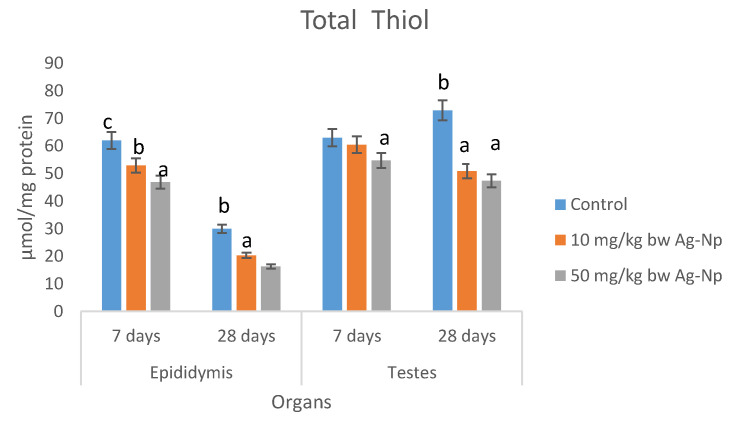
Effects of sub-dermal administration of Ag-NPs on total thiol levels in the testis and epididymis of rats. Each bar represent mean ± standard deviation of triplicate determination. Bars with different superscript alphabet are significantly different (*p* < 0.05). The high significant levels of the parameters were in the order of a < b < c. Data with superscript alphabet “a” are significantly lower than data with superscript alphabet “b” while data with superscript “b” are lower than data with superscript alphabet “c” at *p* < 0.05.

**Figure 5:**
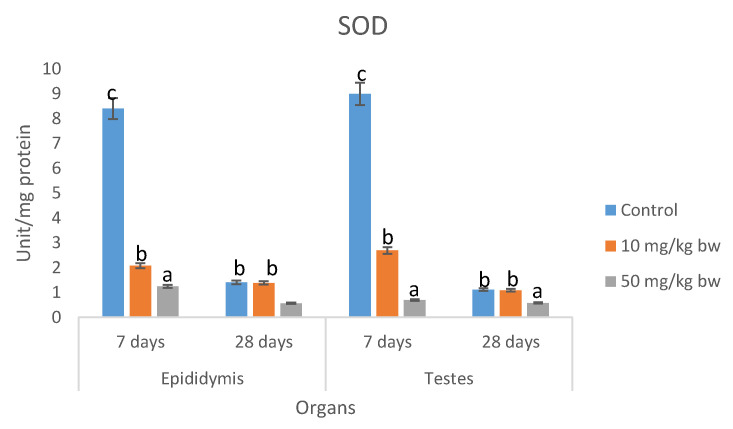
Effects of sub-dermal administration of Ag-NPs on SOD activities in the testis and epididymis of rats. Each bar represent mean ± standard deviation of triplicate determination. Bars with different superscript alphabet are significantly different (*p* < 0.05). The high significant levels of the parameters were in the order of a < b < c. Data with superscript alphabet “a” are significantly lower than data with superscript alphabet “b” while data with superscript “b” are lower than data with superscript alphabet “c” at *p* < 0.05.

**Figure 6:**
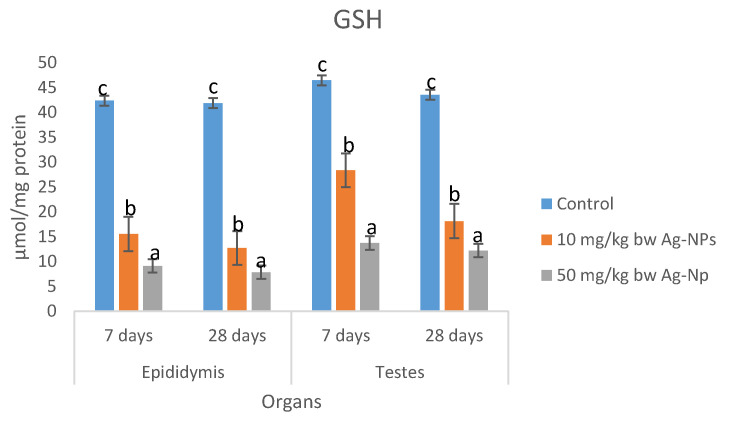
Effects of sub-dermal administration of Ag-NPs on GSH activity in the testis and epididymis of rats. Each bar represent mean ± standard deviation of triplicate determination. Bars with different superscript alphabet are significantly different (*p* < 0.05). The high significant levels of the parameters were in the order of a < b < c. Data with superscript alphabet “a” are significantly lower than data with superscript alphabet “b” while data with superscript “b” are lower than data with superscript alphabet “c” at *p* < 0.05.

**Figure 7:**
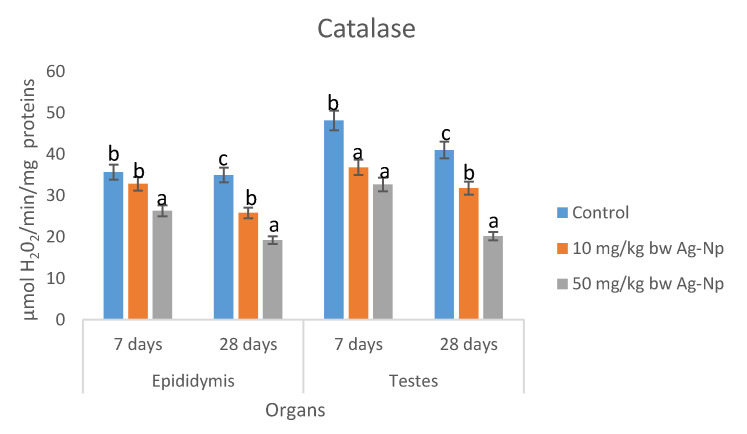
Effects of sub-dermal administration of Ag-NPs on catalase activity in the testis and epididymis of rats. Each bar represent mean ± standard deviation of triplicate determination. Bars with different superscript alphabet are significantly different (*p* < 0.05). The high significant levels of the parameters were in the order of a < b < c. Data with superscript alphabet “a” are significantly lower than data with superscript alphabet “b” while data with superscript “b” are lower than data with superscript alphabet “c” at *p* < 0.05.

There was a dose-dependent decrease in the concentrations of total proteins in epididymis and testes of rats; dosed Ag-NP for 7 and 28 days. There were no significant differences (*p* > 0.05) in the concentrations of total proteins in epididymis between the Ag-NP treated groups and the control group (Table 8). The concentrations of lipid peroxidation (Table 9) and H_2_O_2_ (Table 10) in epididymis and testes of rats dosed 50 mg/kg bw were significantly higher than the control groups; however, the concentrations in rats dosed 10 mg/kg bw Ag-NP compared well (*p* < 0.05) with the controls. There were dose-dependent significant decreases in activities of superoxide dismutase (SOD) catalase, reduced glutathione (GSH), and total thiol groups in epididymis and testes of rats dosed Ag-NP for 7 and 28 days (Figure 4, Figure 5, Figure 6 and Figure 7).

### 2.8. Histopathology

**Figure 8:**
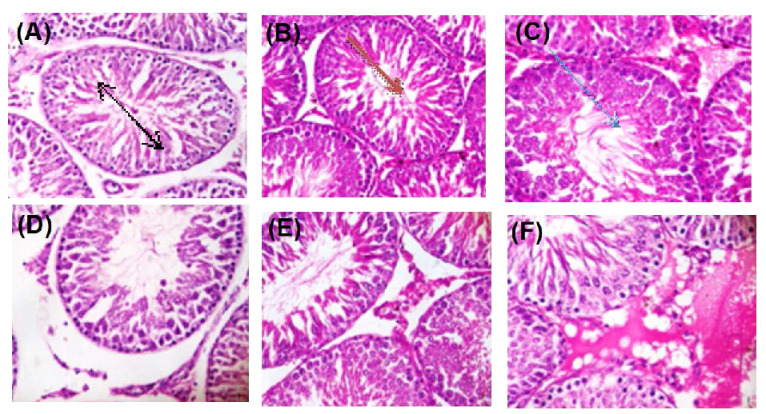
Photomicrograph of testes sections after exposure of rats to Ag-NPs showing; (**A**) several normal seminiferous tubules with normal spermatogonia cell, normal Sertoli cells, normal germ cell layer with normal maturation stages; (**B**) interstitial spaces show an area of interstitial congestion; (**C**) the lumen devoid of spermatozoa with interstitial congestion; (**D**) normal seminiferous tubules with normal spermatogonia cell, normal Sertoli cells, and normal germ cell layer; (**E**) maturation arrest of tubules and mild interstitial congestion; (**F**) seminiferous tubules with atrophy exhibiting thick double cell layers indicative of cessation of spermatogenesis. Magnification 400×. A: Control, B: 10 mg/kg bw, C: 50 mg/kg bw, D: Control, E: 10 mg/kg bw, F: 50 mg/kg bw. A–C (7 days exposure of Ag-NP), D–F (28 days exposure of Ag-NP).

**Figure 9:**
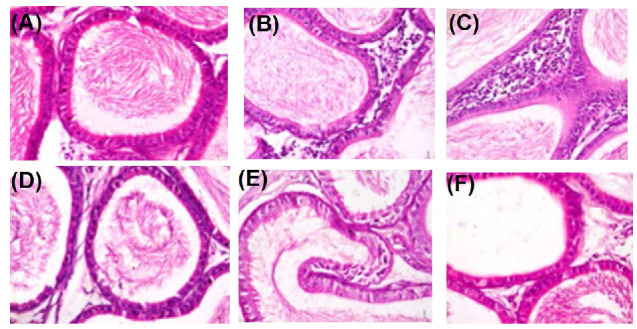
Photomicrograph of epididymis after exposure of rats to Ag-NPs showing (**A**) epididymal ducts with normal smooth muscle layer and epithelial layers, stored content of spermatozoa within the lumen; (**B**) interstitial spaces with moderately increased interstitial connective tissues; (**C**) mildly increased fibrotic tissues, interstitial spaces with severe infiltration of connective tissues and inflammatory cells; (**D**) normal smooth muscle layer and epithelial layers; (**E**) interstitial spaces with moderate infiltration of inflammatory cells and interstitial congestion; (**F**) interstitial congestion with ducts having empty lumen lacking spermatozoa. Magnification 400×. A: Control, B: 10 mg/kg bw, C: 50 mg/kg bw, D: Control, E: 10 mg/kg bw, F: 50 mg/kg bw. A–C (7 days exposure of Ag-NP), D–F (28 days exposure of Ag-NPs).

Administration of Ag-NP to rats at 50 mg/kg bw caused degenerative alterations to the cellular architecture of rat testes (Figure 8) and epididymis (Figure 9) relative to the control.

## 3. Discussion

The aggregation state of Ag-NP is an important property to evaluate since it impacts NP fate, transport, and toxicity [16]. In Figure 1 and Figure 2, the SEM and TEM micrographs revealed that Ag-NP formed small, loosely packed aggregates. Furthermore, the TEM micrograph indicated that aggregation of the primary Ag-NP in the aqueous phase was more pronounced compared to the dry form; consequently, indicating an increase in Ag-NP size when in suspension. The high negative potential value indicates long term stability, good colloidal nature, and high dispersity of AgNPs due to negative–negative repulsion [17]. The Ag-NPs surface charges could determine the toxicity effect in cells. However, the negative surface charge of the Ag-NPs renders them unsuitable, cause of its strong affinity for murine red blood cells compared to positively-charged NPs [18].

The negative influence of toxic compounds, xenobiotic on the bodyweight of the laboratory animal species is recognized and well-documented in published pieces of literature. Previous studies have presented a contradictory report on the effect of nanoparticles on bodyweight. The decreases in bodyweight of rats observed in this study could be attributed to altered physiological process which probably affects the animal’s appetite and feeds consumption with consequent effects on the body weight. DeJong et al. [19] reported significant growth retardation in rats after subacute (28 days) intravenous injection of Ag-NP, while Zhang et al. [20] also reported that injection of gold nanoparticles for 10–14 days caused transient reversible changes in bodyweight of the mice. This finding; however, contrary to the findings of Lee et al. [21]; who reported no significant dose-related bodyweight changes during and after Ag-NP, Au-NP, or a combined administration to rats. This discrepancy could be attributed to the particulate size of the NPs [19].

Studies have shown that changes in relative organs and body weight may be a sensitive indicator of the adverse effects of drug/chemicals or toxicants [22,23]. The significant reduction in the relative weight of epididymis and testes in rats dosed Ag-NP for 7 days is an indication that Ag-NP induced atrophy of the epididymis and testes in male rats. Similarly, Watanabe [24] also reported reductions of the relative weights of the seminal vesicle and prostate to bodyweight following 19 days administration of Ag-NP to rats. Previous studies suggested that when Ag-NP and Au-NP are ingested by animals, it circulates in the blood mainly in particulate forms; thus, interacting with blood components and cells to induce coagulative reaction [21,25]. The significant decreases in the levels RBC and Hb of rats dosed 10 and 50 mg/kg bw Ag-NP for 7 days reflect the hematotoxic effects of Ag-NP which could be attributed to the generations of free radicals that compromises the integrity of the membrane [26,27,28,29]. The results of the present study correlate with several reports on the hematotoxic effect of Ag-NPs both in lower and higher animals. In lower animals, Shaluei et al. [30] reported decreased levels of RBC and HB levels in silver carp to expose to Ag-NP for 7 days, while Imani et al. [31] reported a decreased level of Hct in rainbow trout after 8 days of Ag-NP administration. Similarly, in higher animals, Cheraghi et al. [32] reported decreased levels of RBC and HCT following 15 days of oral dosing of rats with Ag-NP. It is noteworthy that the hematotoxic effect of Ag-NP in the present study and those reported in several works of literature were reported under the observation of 2 weeks or less. The 28-day administration exerts a reversal effect on RBC and Hb levels of the animal, thus demonstrating hematopoietic properties. The significant decrease in MPV, MCH, and MCV in rats dosed 50 mg/kg bw Ag-NP for 7 days, and the significant increases in these parameters in rats dosed 10 mg/kg bw for 28 days is an indication that Ag-NP at higher dose may be hematotoxic but possess hematopoietic properties at a lower dose (10 mg/kg bw). This also further strengthened our earlier claim that Ag-NP produces toxic properties to hematological indices during a short time of administration and beneficial properties upon prolonged administration. The beneficial effect of nanoparticles on hemato-biochemical recovery in disease condition has also been well documented [5,6]. In line with the findings from the present study, Tiwari et al. [33] also reported that intravenously administration of Ag-NP at 10 mg/kg bw produce no toxic effect to hemato-biochemical indices in rats but at 20 mg/kg and above, a significant change in the levels of RBC, WBC, Hb, and platelet counts were noted. The authors also reported that the levels of biomarker enzymes including aspartate transaminase (AST), alanine transaminase (ALT), gamma-glutamyltransferase (GGT) were elevated when 50 mg/kg of Ag-NP was administered. The significant dose-dependent decrease (*p* < 0.05) in WBC, MID, and Lym count reported hereafter the 7 and 28 days of nanoparticle administration could be attributed to increased immunogenic response [34]. Cheraghi et al. [32] also found fewer WBCs in the treated rats. The hematotoxic effect of Ag-NPs observed at higher doses could result in the redistribution of blood flow to the vital organs and decrease testicular blood flow. In line with this study, Koskinen et al. [35] also reported that hypoxia-induced decrease in blood parameters resulted in a 24% decrease in testicular blood flow. These observations must have contributed to the morphological and degenerative changes of testes, epididymis, and spermatogenesis caused by the Ag-NP.

It has been reported that nanoparticles at a dose of 44 µg/mL, accumulate in the sperm tails and head, causing 25% sperm immobility [36]. Similarly, the nanoparticle has been reported to alter testicular morphology and daily sperm production [37]. The dose-dependent decreases in all sperm velocity parameters, sperm total motility, and progressive motility, and the increases in sperm non-progressive motility, immobility, and sperm kinematics parameters following treatment with Ag-NP, compares well with those of Abu et al. [38], Madan [39], Reuben et al. [40], and Obinna and Agu [41] where low testosterone levels in animals impaired spermatogenesis by causing a decrease in sperm count and motility, increase in the percentage of defective sperm cells, and altered histomorphology of testis and/or epididymis. 

The significant (*p* < 0.05) dosed dependent decreases in the hormonal concentrations of LH, FSH, and testosterone in rats dosed Ag-NP for 7 days and 28 days were consistent with previous studies on the effect of different nanoparticles on hormonal levels of male rats. Lafuente et al. [42] reported a significant decrease in the level of testosterone after treatment with ZnO-NP this was; however, reversed by the administration of antioxidants supplement (quercetin). Adebayo et al. [43] also reported a decrease in testosterone, FSH, LH, and prolactin after treatment with CeO_2_-NP. However, the present study contradicts the findings of Mathias et al. [44] who reported that Ag-NP administered to rats at doses of 15 and 30 µg/kg; did not alter the hormonal levels of testosterone, estradiol, FSH, and LH profiles in rats, while Garcia et al. [45] reported increase intra-testicular testosterone level when low-dose (1 mg/kg/dose) Ag-NP was administered intravenously to male mice. Indeed, these discrepancies might be influenced by different factors, such as particle type, size, concentration, and time of exposure [10]. The low levels of LH and FSH might have hampered the secretion of testosterone from the testis with a consequent negative effect on steroidogenesis and spermatogenesis [46]. 

The generation of free radicals and induction of oxidative stress are the well-documented cellular effect of Ag-NP [47]. Ag-NP penetrate the cellular organs particularly the mitochondria which impairs the membrane potential and induces the production of free radicals, this is evident by the elevated levels of H_2_O_2_ and MDA, and the reduction of the activities of antioxidant enzymes, especially catalase, SOD, and GSH observed in testes and epididymis of rats dosed Ag-NP for 7–28 days. The decreased levels of catalase, SOD, and GSH after exposure to silver nanoparticles may be due to complexing of silver nanoparticles with thiol groups [48,49] or to the increasing use of GSH, catalase, and SOD to downplay the effect of free radicals after exposure to of the nanoparticles [50].

SOD and CAT are antioxidant enzymes that protect the body against free radicals and oxidative stress. SOD is a first-line enzymatic defense enzyme that catalyzed the dismutation of superoxide anion to hydrogen peroxide and water molecules. CAT and GPX are considered the second line defense system that acts upon the product of SOD activities (H_2_O_2_) to produce harmless molecular oxygen and water molecules [51,52,53]. The results of the present study suggest that the animal antioxidant defense ability was depressed and that the integrity of testes and epididymis has been compromised by Ag-NP, resulting in the accumulation of reactive oxygen species (ROS) and lipid peroxidation which in turn induces oxidative stress in the testes and epididymis tissue. Similar studies reported that Ag-NP decreased CAT activities and increased MDA levels in the liver of fish [54] and rats [55], which resulted in oxidative damage of the liver. Moreover, in a study by Paio et al. [56], it was shown that Ag-NP induced oxidative stress damage in the human liver by inhibiting glutathione reduction and inducing mitochondrial-dependent cell death. Adeyemi and Faniyan [57] have proposed that the reduction of catalase activity may be related to the interaction of Ag-NP with thiol groups of this enzyme.

Administration of Ag-NP to rats at 50 mg/kg bw caused degenerative alterations to the cellular architecture of rat testes and epididymis relative to the control. These degenerations caused by the nanoparticle are lines of evidence supporting the potential of the nanoparticle to cause cellular and oxidative damage to rats’ testes and epididymis. Previous studies have shown the potential of nanoparticles to confiscate in tissues and induced cellular damage. The findings of the present study, therefore, showed that the Ag-NP could significantly alter the fertility potential of male rats.

## 4. Materials and Method

### 4.1. Synthesis and Characterization of Silver Nanoparticle (Ag-NP)

Exactly, 0.8 mL of AgNO_3_ (0.5 M) was mixed well with aqueous 0.4 M N,N-dimethylformamide (3 mL), and the resulting solution was hydrothermally treated at 90 °C for 2 h. Finally, the reduction of Ag+ ions were monitored by measuring the UV–visible spectrum of the solution on a spectrophotometer (PerkinElmer Spectrum 400, Waltham, MA, USA). At first, the Ag-NP was dissolved in phosphate buffered saline and then made to a final concentration of 10 and 50 mg/kg [13]. Scanning electron microscopy (SEM) analysis of synthesized Ag-NPs was done using a JSM-7500F, SEM machine (Waltham, MA, USA). The size and shape of the synthesized AgNPs were determined by transmission electron microscopy (TEM) (JEOL, Peabody, MA, USA). The zeta potential of the Ag-NP was measured using a Zetasizer Nano ZS instrument (Malvern, Worcestershire, UK). The surface area was estimated using a Brunauer–Emmett–Teller analyzer (Nova 3200e, Boynton Beach, FL, USA).

### 4.2. Experimental Animals

A total of thirty (30) rats weighing between 100–150 g were purchased from the Animal House Unit, University of Ibadan, Nigeria. They were kept in clean metabolic cages placed in a well-ventilated house condition (Temperature 23 ± 1 °C: Photoperiod: 12 h light and 12 h dark cycle each throughout the experimental period; humidity: 45–50%). All animal experiments were carried out in accordance with the UK Animals (Scientific Procedures) Act, 1986 and associated guidelines, the European communities’ council directive of 24 November 1986 (86/609/EEC) and the National Institute of Health guide for the care and use of laboratory animals (NIH Publications No. 8023, revised 1978). The principles governing the use of laboratory animals as laid out by the Bingham University, Committee on Ethics for Medical and Scientific Research were duly observed.

### 4.3. Experimental Design

The rats were divided into 6 groups (A–F) of 5 rats each. Groups B and E were treated with 10 and 50 mg/kg bw Ag-NP, Groups C and F were treated with 10 and 50 mg/kg bw Ag-NP. Groups A and D were set up as normal control and were given 0.2 mL normal saline for 7 and 28 days respectively. Group B and C were treated with Ag-NP for 7 days while Group E and F were treated with Ag-NP for 28 days.

### 4.4. Sample Collection and Preparation

After treatment with Ag-NP, the animals were fasted overnight and sacrificed under anesthesia and blood was collected in EDTA bottle for hematological analysis. Another set of blood was collected in EDTA free sample bottle, the blood was allowed to clot and centrifuge at 3000 rpm for 10 min to obtain the serum which was collected and kept in a freezer (−20 °C) until needed for hormonal assays. The epididymis and testes were identified, removed, cleared of fat, and weighed. Semen was then collected by incising of the cauda epididymis of each animal on a clean glass slide and sperm parameters were analyzed using a computer-assisted sperm analyzer (CASA) system (SpermVision™ Minitüb, Tiefenbach, Germany) with Olympus BX 51 phase contrast microscope (Olympus, Tokyo, Japan) based on WHO5 new edition guidelines.

### 4.5. Evaluation of Hematological Parameters.

The hematological components including Hb, PCV, RBC, PLT, MCV, MCH, MCHC, and total and differential WBC were determined using the automated hematologic analyzer SYSMEX KX21 (SYSMEX Corporation, Kobe, Japan) employing the principle described by Dacie and Lewis [58].

### 4.6. Hormonal Analysis

Luteinizing hormone (LH), follicle-stimulating hormone (FSH), and testosterone were determined according to the method described by Schwarzstein et al. [59].

### 4.7. Analysis of Testes and Epididymis Biochemical Parameters

The testes and epididymis were homogenized in phosphate buffer (pH 7.4) and centrifuged at 10,000 net grams for 10 min at 4 °C and the supernatants were used for estimation biochemical parameters using standard protocols. Lipid peroxidation was determined by measuring the thiobarbituric acid reactive substance (TBARS) as described by Varshney and Kale [60]. Total thiol as described by Hu and Dillard [61]. Protein concentrations by the method of Gornal et al. [62], catalase activity by the method of Sinha [63], SOD by the method of Misra and Fridovich [64], and GSH as described by Beutler et al. [65].

### 4.8. Histological Procedures

Following the collection of testicular samples, the organs were promptly fixed in Bouins fluid to preserve the structure and molecular composition of the testes. Further histological preparations were carried out as described by Igwebuike and Eze [66] and stained with hematoxylin and eosin for light microscopy. 

### 4.9. Data Analysis

Data analysis was performed using SPSS (version 21.0; SPSS Inc., Chicago, IL, USA). Comparisons between different groups were carried out using one-way analysis of variance (ANOVA) followed by Duncan multiple range test (DMRT). Data were expressed as mean ± SD of triplicate determinations. Significant was considered at *p* < 0.05.

## Figures and Tables

**Table 1:** Weight changes of male rats following administration of Ag-NP.

	Initial Body Weight (g)	7 Days Final Body Weight (g)	Weight Gain/Loss	Initial Body Weight (g)	28 Days Final Body Weight (g)	Weight Gain/Loss
Control	120.60 ± 12.24	145.50 ± 20.91	24.90 ± 3.45 ^b^	137.80 ± 4.40	170.50 ± 11.18	32.70 ± 5.43 ^c^
10 mg/kg bw	150.45 ± 17.67	132.20 ± 8.72	−18.25 ± 4.35 ^a^	175.45 ± 17.67	154.80 ± 7.85	−20.65 ± 3.45 ^a^
50 mg/kg bw	165.32 ± 13.69	146.20 ± 7.00	−19.12 ± 2.57 ^a^	205.55 ± 20.90	170.80 ± 4.80	−34.75 ± 4.87 ^b^

Data expressed as mean ± standard deviation of triplicate determination. Data followed by different superscript alphabet along the same column are significantly different (*p* < 0.05). The high significant levels of the parameters were in the order of a < b < c. Data with superscript alphabet “a” are significantly lower than data with superscript alphabet “b” while data with superscript “b” are lower than data with superscript alphabet “c” at *p* < 0.05.

**Table 2:** Relative weight of organs after administration of Ag-NP to male rats.

	Epididymis (g)		Testes (g)	
	7 Days	28 Days	7 Days	28 Days
Control	3.47 ± 1.19 ^b^	3.15 ± 0.92 ^a^	4.01 ± 1.51 ^b^	3.35 ± 0.86 ^a^
10 mg/kg bw	2.47 ± 1.03 ^a^	2.96 ± 0.76 ^a^	2.78 ± 0.96 ^a^	3.75 ± 1.10 ^a^
50 mg/kg bw	2.89 ± 0.97 ^a^	3.25 ± 1.03 ^a^	3.21 ± 1.49 ^a,b^	3.95 ± 1.513 ^a^

Data expressed as mean ± standard deviation of triplicate determination. Data followed by different superscript alphabet along the same column are significantly different (*p* < 0.05). The high significant levels of the parameters were in the order of a < b. Data with superscript alphabet “a” are significantly lower than data with superscript alphabet “b” at *p* < 0.05.

**Table 3:** Effects of sub-dermal administration of Ag-NP on hematological parameters in rats.

		7 Days			28 Days	
	Control	10 mg/kg	50 mg/kg	Control	10 mg/kg	50 mg/kg
WBC (×10^3^/mm^3^)	11.77 ± 3.39 ^c^	10.68 ± 7.51 ^b^	6.23 ± 0.59 ^a^	6.18 ± 2.38 ^b^	5.99 ± 1.75 ^ab^	5.86 ± 2.81 ^a^
NEU (%)	10.54 ± 3.31 ^c^	5.67 ± 0.29 ^b^	3.41 ± 0.18 ^a^	3.14 ± 0.97 ^b^	3.21 ± 0.59 ^b^	2.167 ± 0.40 ^a^
MID (%)	0.96 ± 0.46 ^c^	0.87 ± 0.16 ^b^	0.63 ± 0.34 ^a^	0.61 ± 0.12 ^b^	0.57 ± 0.09 ^a^	0.67 ± 0.37 ^a^
GRA	0.27 ± 0.11 ^b^	0.23 ± 0.04 ^b^	0.13 ± 0.09 ^a^	0.07 ± 0.03 ^a^	0.21 ± 0.13 ^b^	0.20 ± 0.16 ^b^
LYM (%)	89.07 ± 4.30 ^c^	77.90 ± 1.21 ^b^	47.00 ± 2.6 ^a^	46.00 ± 3.6 ^b^	76.23 ± 1.28 ^b^	39.47 ± 4.19 ^c^
MID (%)	14.13 ± 0.40 ^b^	11.43 ± 2.22 ^a^	10.97 ± 2.6 ^a^	12.17 ± 3.1 ^b^	9.77 ± 1.47 ^a^	8.23 ± 4.70 ^a^
GRA (%)	3.43 ± 0.50 ^a^	3.17 ± 0.23 ^a^	5.60 ± 7.10 ^a^	3.43 ± 1.40 ^b^	1.91 ± 0.31 ^a^	3.40 ± 0.46 ^b^
RBC (10^12^L)	6.75 ± 0.13 ^a^	6.56 ± 1.19 ^a^	6.29 ± 2.85 ^a^	5.56 ± 1.47 ^a^	6.15 ± 0.73 ^b^	7.06 ± 1.79 ^c^
HB (g/dl)	161.33 ± 8.1 ^b^	156.0 ± 13.9 ^b^	134.00 ± 32 ^a^	128.3 ± 12.22 ^a^	138.33 ± 7.3 ^b^	153.67 ± 10.45 ^c^
MCV (f/l)	74.27 ± 1.42 ^b^	77.67 ± 1.53 ^b^	52.73 ± 0.51 ^a^	54.30 ± 1.66 ^a^	86.00 ± 1.00 ^b^	55.53 ± 1.76 ^a^
MCH (pg)	30.0 ± 0.50 ^b^	29.50 ± 0.78 ^b^	15.60 ± 3.1 ^a^	25.67 ± 4.38 ^a,b^	28.17 ± 0.73 ^b^	22.57 ± 5.16 ^a^
RDW-SD	32.30 ± 1.91 ^b^	30.03 ± 4.30 ^b^	25.40 ± 2.2 ^a^	26.27 ± 2.40 ^a^	28.43 ± 3.99 ^a^	29.97 ± 2.30 ^a^
RDW-CV	17.17 ± 0.25	15.70 ± 0.40 ^a^	15.30 ± 0.49 ^a^	13.60 ± 2.16 ^a^	14.73 ± 0.31 ^a^	14.17 ± 2.75 ^a^
PLT (10^3^ µL)	286.00 ± 9.5 ^a^	305.0 ± 5.00 ^b^	261.33 ± 10 ^a^	272.33 ± 5.5 ^a^	334.67 ± 12 ^b^	296.67 ± 4 ^a^
MPV (g/dl)	7.49 ± 0.08 ^b^	7.30 ± 0.10 ^b^	6.83 ± 0.15 ^a^	6.67 ± 0.61 ^a^	7.27 ± 0.15 ^b^	6.63 ± 0.32 ^a^
PCW	9.90 ± 0.10 ^a^	10.20 ± 0.12 ^a^	14.0 ± 2.21 ^b^	12.57 ± 0.40 ^b^	10.50 ± 0.61 ^a^	12.93 ± 1.10 ^b^
HCT	0.21 ± 0.01 ^a^	0.22 ± 0.01 ^a^	0.27 ± 0.17 ^a^	0.36 ± 0.11 ^a^	0.34 ± 0.03 ^a^	0.35 ± 0.07 ^a^

Data expressed as mean ± standard deviation of triplicate determination. Data followed by different superscript alphabet along the same column are significantly different (*p* < 0.05). The high significant levels of the parameters were in the order of a < b < c. The high significant levels of the parameters were in the order of a < b < c. Data with superscript alphabet “a” are significantly lower than data with superscript alphabet “b” while data with superscript “b” are lower than data with superscript alphabet “c” at *p* < 0.05. WBC: white blood cell count; RBC: red blood cell count; Hb: hemoglobin concentration; HCT: hematocrit; MCV: mean corpuscular volume; MCH: mean corpuscular hemoglobin; MCHC: mean corpuscular hemoglobin concentration; RDW: red cell distribution width; PLT: platelet count; MPV: mean platelet volume; LYM%: percent of lymphocytes; NEU: neutrophil; MID: mixed.

**Table 4:** Effects of sub-dermal administration of Ag-NP on LH, FSH, and testosterone of experimental rats.

	LH (mIU/mL)		FSH (mIU/mL)		Testosterone (ng/mL)	
	7 Days	28 Days	7 Days	28 Days	7 Days	28 Days
Control	1.2 ± 0.22 ^c^	0.7 ± 0.3 ^c^	2.8 ± 0.24 ^c^	1.10 ± 0.21 ^c^	3.10 ± 0.51 ^c^	2.40 ± 0.53 ^c^
10 mg/kg bw	0.8 ± 0.32 ^b^	0.6 ± 0.2 ^b^	1.2 ± 0.21 ^b^	0.89 ± 0.10 ^b^	2.80 ± 0.19 ^b^	1.00 ± 0.51 ^b^
50 mg/kg bw	0.69 ± 0.4 ^a^	0.5 ± 0.22 ^a^	0.9 ± 0.6 ^a^	0.75 ± 0.12 ^a^	2.20 ± 0.71 ^a^	0.52 ± 0.23 ^a^

Data expressed as mean ± standard deviation of triplicate determination. Data followed by different superscript alphabet along the same column are significantly different (*p* < 0.05). The high significant levels of the parameters were in the order of a < b < c. Data with superscript alphabet “a” are significantly lower than data with superscript alphabet “b” while data with superscript “b” are lower than data with superscript alphabet “c” at *p* < 0.05.

**Table 5:** Effects of sub-dermal administration of Ag-NP on sperm motility in male rats.

	Total Motility (%)	Progressive Motility (%)	Non-Progressive (%)	Immobility (%)
	7-day treatment			
Control	60.29 ± 5.78 ^c^	62.02 ± 5.46 ^c^	30.34 ± 3.57 ^a^	35.61 ± 1.91 ^a^
10 mg/kg	40.71 ± 3.35 ^b^	34.44 ± 3.97 ^b^	44.36 ± 8.60 ^b^	56.24 ± 2.53 ^b^
50 mg/kg	20.23 ± 1.69 ^a^	9.20 ± 0.69 ^a^	46.19 ± 4.49 ^b^	66.49 ± 4.10 ^c^
	28-day treatment			
Control	57.16 ± 4.49 ^c^	62.76 ± 5.33 ^c^	44.29 ± 9.12 ^a^	29.99 ± 5.24 ^a^
10 mg/kg	34.13 ± 0.57 ^b^	25.10 ± 6.52 ^b^	52.76 ± 4.71 ^b^	63.43 ± 2.90 ^b^
50 mg/kg	9.65 ± 0.53 ^a^	3.88 ± 1.33 ^a^	59.05 ± 0.51 ^b^	76.81 ± 4.31 ^c^

Data expressed as mean ± standard deviation of triplicate determination. Data followed by different superscript alphabet along the same column are significantly different (*p* < 0.05). The high significant levels of the parameters were in the order of a < b < c. Data with superscript alphabet “a” are significantly lower than data with superscript alphabet “b” while data with superscript “b” are lower than data with superscript alphabet “c” at *p* < 0.05.

**Table 6:** Effects of sub-dermal administration of Ag-NP on sperm kinematics in male rats.

	Straight-Line Velocity (µm/s)	Curvilinear Velocity (µm/s)	Average Path Velocity (µm/s)
	7-day treatment		
Control	6.22 ± 0.16 ^c^	19.52 ± 2.25 ^c^	12.54 ± 0.57 ^c^
10 mg/kg bw	2.15 ± 0.10 ^b^	3.14 ± 0.35 ^b^	3.02 ± 0.59 ^b^
50 mg/kg bw	0.74 ± 0.06 ^a^	0.70 ± 0.27 ^a^	1.46 ± 0.47 ^a^
	28-day treatment		
Control	4.77 ± 0.49 ^c^	21.74 ± 2.64 ^b^	0.43 ± 0.08 ^a^
10 mg/kg bw	1.84 ± 0.52 ^b^	1.84 ± 0.52 ^a^	0.63 ± 0.21 ^b^
50 mg/kg bw	0.43 ± 0.08 ^a^	2.10 ± 0.08 ^a^	0.61 ± 0.13 ^b^

Data expressed as mean ± standard deviation of triplicate determination. Data followed by different superscript alphabet along the same column are significantly different (*p* < 0.05). The high significant levels of the parameters were in the order of a < b < c. Data with superscript alphabet “a” are significantly lower than data with superscript alphabet “b” while data with superscript “b” are lower than data with superscript alphabet “c” at *p* < 0.05.

**Table 7:** Effects of sub-dermal administration of Ag-NP on sperm velocity parameters in male rats.

	ALH (µm)	Beat Cross Frequency (Hz)	Linearity (%)	Straightness (%)
	7-day treatment			
Control	4.09 ± 0.61 ^c^	6.80 ± 0.52 ^c^	56.75 ± 8.26 ^c^	61.35 ± 4.33 ^c^
10 mg/kg bw	0.91 ± 0.04 ^b^	3.40 ± 0.18 ^b^	34.18 ± 4.34 ^b^	42.69 ± 1.41 ^b^
50 mg/kg bw	0.61 ± 0.13 ^a^	1.52 ± 0.20 ^a^	22.88 ± 4.57 ^a^	7.17 ± 0.93 ^a^
	28-day treatment			
Control	6.14 ± 1.33 ^c^	8.09 ± 1.45 ^c^	66.46 ± 9.50 ^c^	88.08 ± 11.94 ^c^
10 mg/kg bw	0.63 ± 0.22 ^b^	4.20 ± 0.55 ^b^	30.95 ± 1.65 ^b^	30.93 ± 1.13 ^b^
50 mg/kg bw	0.33 ± 0.09 ^a^	2.61 ± 0.41 ^a^	10.15 ± 0.70 ^a^	7.24 ± 1.62 ^a^

Data expressed as mean ± standard deviation of triplicate determination. Data followed by different superscript alphabet along the same column are significantly different (*p* < 0.05). The high significant levels of the parameters were in the order of a < b < c. Data with superscript alphabet “a” are significantly lower than data with superscript alphabet “b” while data with superscript “b” are lower than data with superscript alphabet “c” at *p* < 0.05.

**Table 8:** Effects of sub-dermal administration of Ag-NP on protein concentrations in testes and epididymis experimental rats.

Proteins	Epididymis (g/dL)		Testes (g/dL)	
	7 Days	28 Days	7 Days	28 Days
Control	1.41 ± 0.32 ^c^	1.07 ± 0.36 ^c^	1.52 ± 0.04 ^a^	1.26 ± 0.01 ^a^
10 mg/kg bw	0.89 ± 0.03 ^b^	0.93 ± 0.39 ^b^	1.02 ± 0.02 ^a^	1.20 ± 0.02 ^a^
50 mg/kg bw	0.21 ± 0.03 ^a^	0.27 ± 0.0 ^a^	1.08 ± 0.02 ^a^	1.23 ± 0.05 ^a^

Data expressed as mean ± standard deviation of triplicate determination. Data followed by different superscript alphabet along the same column are significantly different (*p* < 0.05). The high significant levels of the parameters were in the order of a < b < c. Data with superscript alphabet “a” are significantly lower than data with superscript alphabet “b” while data with superscript “b” are lower than data with superscript alphabet “c” at *p* < 0.05.

**Table 9:** Effect of sub-dermal administration of Ag-NP on malondialdehyde (MDA) level in testes and epididymis of experimental rats.

MDA (Units/g Tissue × 10^6^)	Epididymis (Unit/g Tissue × 10^6^)		Testes (Unit/g Tissue × 10^6^)	
	7 Days	28 Days	7 Days	28 Days
Control	17.68 ± 10.13 ^a^	15.42 ± 6.03 ^a^	23.43 ± 2.86 ^a^	16.75 ± 8.30 ^a^
10 mg/kg bw	20.65 ± 9.46 ^a^	16.93 ± 8.80 ^a^	27.34 ± 9.20 ^a^	18.19 ± 3.51 ^a^
50 mg/kg bw	35.49 ± 8.52 ^b^	33.48 ± 5.69 ^b^	36.90 ± 11.10 ^b^	34.09 ± 9.89 ^b^

Data expressed as mean ± standard deviation of triplicate determination. Data followed by different superscript alphabet along the same column are significantly different (*p* < 0.05). The high significant levels of the parameters were in the order of a < b < c. Data with superscript alphabet “a” are significantly lower than data with superscript alphabet “b” while data with superscript “b” are lower than data with superscript alphabet “c” at *p* < 0.05.

**Table 10:** Effects of sub-dermal administration of Ag-NP on H_2_O_2_ generation in testes and epididymis of experimental rats.

H_2_O_2_(μmol/mg Protein)	Epididymis		Testes	
	7 Days	28 Days	7 Days	28 Days
Control	33.95 ± 0.37 ^a^	37.1 ± 0.29 ^a^	34.63 ± 0.34 ^a^	34.5 ± 0.40 ^a^
10 mg/kg bw	34.65 ± 0.24 ^a^	38.75 ± 0.32 ^a^	36.66 ± 0.24 ^ab^	36.4 ± 0.57 ^a^
50 mg/kg bw	38.05 ± 0.33 ^a^	40.13 ± 0.60 ^b^	38.95 ± 0.21 ^b^	39.78 ± 0.40 ^b^

Data expressed as mean ± standard deviation of triplicate determination. Data followed by different superscript alphabet along the same column are significantly different (*p* < 0.05). The high significant levels of the parameters were in the order of a < b < c. Data with superscript alphabet “a” are significantly lower than data with superscript alphabet “b” while data with superscript “b” are lower than data with superscript alphabet “c” at *p* < 0.05.
