# Correction: Olugbodi et al. Silver Nanoparticles Stimulates Spermatogenesis Impairments and Hematological Alterations in Testis and Epididymis of Male Rats. *Molecules* 2020, *25*, 1063

**DOI:** 10.3390/molecules30040858

**Published:** 2025-02-13

**Authors:** Janet Olayemi Olugbodi, Oladipupo David, Ene Naomi Oketa, Bashir Lawal, Bamidele Joseph Okoli, Fanyana Mtunzi

**Affiliations:** 1 Department of Biochemistry, Bingham University, Abuja-Keffi Expressway Road, P.M.B 005 Karu, Nigeria; oketanaomiene87@gmail.com; 2 Department of Medical Bioscience, University of the Western Cape, Bellville, Cape Town 7530, South Africa; 3681075@myuwc.ac.za; 3 PhD Program for Cancer Molecular Biology and Drug Discovery, College of Medical Science and Technology, Taipei Medical University and Academia Sinica, Taipei 111, Taiwan; bashirlawal12@gmail.com; 4 Institute of Chemical and Biotechnology, Vaal University of Technology, Science Park, Private Bag x021, South Africa; okolibj@binghamuni.edu.ng; 5 Department of Chemical Sciences, Bingham University, Abuja-Keffi Expressway Road, P.M.B 005 Karu, Nigeria; 6 DIHLARE Remedy, Faculty of Applied and Computer Sciences, Vaal University of Technology, Science Park, Private Bag x021, South Africa; fanyana@vut.ac.za

## **Error in Figure** 

In the original publication [1], there were mistakes in Figure 1 as published. The scale bars, as well as the figure panel numbers, were missing in Figure 1. In addition, the left panel was presented twice. The corrected Figure 1, with the appropriate scale bars, panel labels, and updated figure caption, appears below.

## **Text Correction** 

There was an error in the original publication [1]. A correction has been made to Section 2.1, in the first sentence: 

The synthesized Ag-NPs were approximately spherical with an average particle size of 113.389 ± 22.964 nm (Figure 1) and a surface area of 7.5329 m^2^/g.

The authors state that the scientific conclusions are unaffected. This correction was approved by the Academic Editor. The original publication has also been updated.

## Figures and Tables

**Figure 1:**
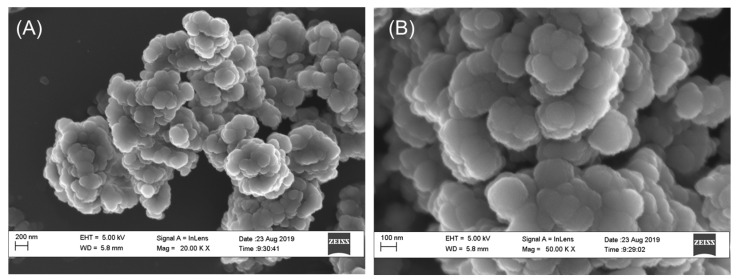
SEM micrographs of dry Ag-NPs at (**A**) 20,000×, and (**B**) 50,000×. The synthesized Ag-NPs were agglomerated and approximately spherical in shape.

## References

[1] Olugbodi J.O., David O., Oketa E.N., Lawal B., Okoli B.J., Mtunzi F. (2020). Silver Nanoparticles Stimulates Spermatogenesis Impairments and Hematological Alterations in Testis and Epididymis of Male Rats. Molecules.

